# Clinical Benefits From Administering Probiotics to Mechanical Ventilated Patients in Intensive Care Unit: A PRISMA-Guided Meta-Analysis

**DOI:** 10.3389/fnut.2021.798827

**Published:** 2022-01-27

**Authors:** Hongzhuan Song, Wenqing Hu, Xiujie Zhou, Jiaping Tao, Siyi Zhang, Xuhong Su, Wenjun Wu

**Affiliations:** ^1^Department of Hematology, Haining People's Hospital, Haining, China; ^2^Department of Emergency, Haining People's Hospital, Haining, China; ^3^Department of Hematology, First Affiliated Hospital, Zhejiang University School of Medicine, Hangzhou, China

**Keywords:** ventilator-associated pneumonia, probiotics, meta-analysis, intensive care unit, mechanical ventilation, critical care, randomized control trial (RCT)

## Abstract

**Background:**

The use of probiotics has been considered as a new intervention for ventilator-associated pneumonia (VAP) prevention in the intensive care unit (ICU). The aim of this meta-analysis was to evaluate the effect of probiotics on mechanical-ventilated patients in ICU.

**Methods:**

PubMed, Embase, Scopus, and the Cochrane Library were searched for relevant randomized controlled trials (RCTs) from their respective inception through October 10, 2021. All studies meeting the inclusion criteria were selected to evaluate the effect of probiotics on patients receiving mechanical ventilation in ICU.

**Results:**

A total of 15 studies involving 4,693 participants met our inclusion criterion and were included in this meta-analysis. The incidence of VAP in the probiotic group was significantly lower (odds ratio [*OR*] 0.58, 95% *CI* 0.41 to 0.81; *p* = 0.002; *I*^2^ = 71%). However, a publication bias may be present as the test of asymmetry was significant (*p* = 0.007). The probiotic administration was associated with a significant reduction in the duration of mechanical ventilation (mean difference [*MD*] −1.57, 95% *CI* −3.12 to −0.03; *p* = 0.05; inconsistency [*I*]^2^ = 80%), length of ICU stay (MD −1.87, 95% *CI* −3.45 to −0.28; *p* = 0.02; *I*^2^ = 76%), and incidence of bacterial colonization (*OR* 0.59, 95% *CI* 0.45 to 0.78; *p* = 0.0001; *I*^2^ = 34%). Moreover, no statistically significant differences were observed regarding the incidence of diarrhea (*OR* 0.90, 95% *CI* 0.65 to 1.25; *p* = 0.54; *I*^2^ = 12%) and mortality (*OR* 0.91, 95% *CI* 0.80 to 1.05; *p* = 0.19; *I*^2^ = 0%) between probiotics group and control group.

**Conclusion:**

Our meta-analysis shows that probiotics are associated with a reduction in VAP, as well as the duration of mechanical ventilation, ICU length of stay, and bacterial colonization, but no significant effects on ICU mortality and occurrence of diarrhea. However, in consideration of the significant heterogeneity and publication bias, our findings need to be further validated.

**Systematic Review Registration:**

https://www.crd.york.ac.uk/prospero/, identifier: CRD42020150770.

## Introduction

Ventilator-associated pneumonia (VAP) is defined as an infectious inflammatory reaction of the lung parenchyma that occurs after mechanical ventilation for more than 48 h (1), which is a common and severe complication in the intensive care unit (ICU). It is reported that VAP affects between 5 and 40% of patients receiving invasive mechanical ventilation, with large variations depending upon the country, ICU type, and criteria used to identify VAP (2). Despite recent advances in the diagnosis and treatment of VAP, it remains one of the most serious problems in the ICU, with a prolonged duration of mechanical ventilation, increased length of ICU and hospital stays, increased cost, and higher mortality risk (3–5). Although many VAP prevention strategies applications are currently available, some strategies have been challenged and the results of clinical trials are disappointing (2, 6). Consequently, despite the epidemiology and diagnostic criteria for VAP are still controversial, and the interpretation of treatment and prevention is still complicated, it is imperative to find new prevention strategies.

Probiotics are a class of active microorganisms that can produce positive effects in the host when administered at the appropriate dosage (7, 8). They can selectively modulate the growth of the microbiome, inhibit colonization with invasive pathogens, and improve the microecological balance of the host (9). Recent meta-analyses (10–13) indicated that the administration of probiotics could significantly reduce the incidence of VAP. However, several studies have described the potential risks of probiotics such as systemic infections, deleterious metabolic activities, excessive immune stimulation in susceptible individuals, gene transfer, and gastrointestinal side effects (14, 15). Due to the lack of large-scale and high-quality randomized controlled trials (RCTs), whether probiotics have beneficial effects on VAP prevention remains controversial.

Recently, Johnstone et al. completed the largest randomized trial to compare the effect of probiotics on preventing VAP in critically ill patients (16). In this multicenter and pragmatic study concerning 2,650 participates, as compared with placebo, the administration of 1 × 10^10^ colony forming units of *Lactobacillus rhamnosus* GG two times daily for 9 days did not significantly reduce the risk of VAP. Therefore, this meta-analysis aimed to elucidate the latest and most convincing evidence about the effects of probiotics on VAP prevention in ICU patients receiving mechanical ventilation.

## Methods

### Data Sources and Study Selection

This meta-analysis was performed according to the PRISMA statement (17) (checklist in Supplementary Material 1). The study protocol has been registered in PROSPERO (CRD 42020150770). PubMed, Embase, Scopus, and Cochrane Library were searched for eligible studies up to October 10, 2021. Search terms included “ventilator-associated pneumonia,” “VAP,” “probiotics,” and “critically ill” relevant studies were enrolled in the present study. The search was limited to studies published in English. The detailed search strategies were recorded in Supplementary Material 2.

### Eligibility Criteria

Study inclusion criteria were as follows: (1) Population: Critically ill patients receiving mechanical ventilation; (2) Intervention: Probiotics; (3) Comparison: Placebo or no drug infusion; (4) Outcomes: primary outcomes were incidence of VAP and overall mortality (ICU, hospital, 28-day mortality). Secondary outcomes were ICU and hospital length of stay, duration of mechanical ventilation, incidence of diarrhea, and incidence of bacterial colonization; (5) Design: randomized controlled trial.

### Data Extraction and Quality Assessment

Two authors (HS and WH) independently retrieved and extracted relevant studies. The basic characteristics of included studies (first author, years of publication, study design, population, intervention and control methods, and definition of VAP) were recorded in Table 1. Any discrepancies in all phases were ultimately resolved through team consensus. Two authors (WH and XZ) independently assessed the risk of bias according to the Cochrane risk of bias tool (32). The evaluation criteria were based on sample selection, allocation concealment (selection bias), blinding of participants and personnel (performance bias), incomplete outcome data (attrition bias), statistical analysis, and outcome validation, selective reporting, and free of the source of funding (reporting bias) measured the degree of bias, the definition of inclusion and exclusion criteria. They were categorized as low risk, high risk, and unclear risk.

**Table 1:** Characteristics, designs, intervention, and control of the included studies.

**Study and year**	**Design and country**	**N**	**Population**	**Intervention**	**Control**	**Definition of ventilator-associated pneumonia**
Johnstone et al. (16)	Double blinded, multicenter, in Canada, the United States, and Saudi Arabia	2,653	Critically ill adult patients	1 × 10^10^ colony forming units of *Lacticaseibacillus rhamnosus* twice daily for a median of 9-day period	Placebo	New, progressive, or persistent radiographic infiltrate on chest radiograph after at least 2 days of mechanical ventilation, plus any 2 of the following: fever or hypothermia, leukocytosis or leukopenia, and purulent sputum
Mahmoodpoor et al. (18)	Double blinded, multicenter, in Iran	102	Critically ill adult patients	2 capsules of probiotic preparation via feeding tube daily for 14 days. Each capsule contained 10^10^ bacteria consisting of *Lactobacillus, Bifdobacterium* and *Streptococcus thermophiles*	Placebo	Quantitative bronchoalveolar lavage fluid culture
Klarin et al. (19)	No blinded, multicenter, in Sweden	137	Critically ill adult patients	10 ml of a solution containing a total of 10^10^ CFU of *Lactiplantibacillus paraplantarum* in oral care procedure BID, until extubation or ICU discharge	No placebo	Chest radiograph combined with at least three of the other four criteria; a purulent tracheal aspirate; positive culture of tracheal aspirates, fever, leukocytosis or leukopenia
Shimizu et al. (20)	Single blinded, single-center, in Japan	72	Sepsis patients	Yakult BL Seichoyaku (contained 1 × 10^8^ of *Bifidobacterium* and *Lactobacillus* per gram) 3g/day via nasal tube, started within 3 days after admission, continued until oral intake	No placebo	Pneumonia arises after endotracheal intubation
Zeng et al. (21)	No blinded, multicenter, in China	235	Critically ill adult patients	0.5g probiotics capsules (each probiotics capsule contained active *Bacillus subtilis* and *Enterococcus faecalis* of 4.5 × 10^9^/0.25 g and 0.5 × 10^9^/0.25 g) TID, started within 24 h of admission, continued until extubation with a maximum of 14 days	No placebo	Chest radiographs combined with at least two of the following criteria: fever, leukocytosis or leukopenia, purulent tracheal aspirates
Banupriya et al. (22)	No blinded, single-center, in India	142	PICU patients	A capsule 2 billion CFU of *Lactobacillus*, 1 billion CFU of *Bifidobacterium*, and 300 million CFU of *Streptococcus* twice a day via nasogastric tube, started at PICU admission, continued for 7 days	No placebo	Pneumonia developing more than 48 h after endotracheal intubation and initiation of MV
Rongrungruang et al. (23)	No blinded, single-center, in Thailand	150	Critically ill adult patients	8 × 10^9^ CFU of *Lactobacillus casei* for oral care and 8 × 10^9^ CFU of *Lactobacillus casei* via enteral feeding once a day for 28 days	No placebo	Chest radiograph in combination with at least 3 of the following 4 criteria: fever, leukocytosis or leukopenia, purulent tracheal aspirate, a semi quantitative culture of tracheal aspirate samples that was positive for pathogenic bacteria
Oudhuis et al. (24)	No blinded, multicenter, in Netherlands	254	Critically ill adult patients	*Lactiplantibacillus paraplantarum* in a dose of 5 × 10^9^ CFU BID through a nasogastric tube, until ICU discharge, death or extubation	Selective decontamination of the digestive tract	Quantitative culture result in bronchoalveolar lavage fluid
Tan et al. (25)	Single blinded, single-center, in China	52	Adult patients with severe TBI	A total of 109 CFU bacteria a day. Containing 0.5 × 10^8^ *Bifidobacterium longum*, 0.5 × 10^7^ *Lactobacillus bulgaricus*, and 0.5 × 10^7^ *Streptococcus thermophilus* TID via nasogastric tube, started within 48 h after admission, continued for 21 days	No placebo	New or progressive radiographic infiltrate with fever, leukocytosis, leucopenia, or purulent tracheobronchial secretions, and positive semiquantitative cultures of tracheobronchial secretions
Barraud et al. (26)	Double blinded, multicenter, in France	167	Critically ill adult patients	2 × 10^10^ of revivable bacteria (mainly *Lactobacillus rhamnosus*, but also *Lactobacillus casei, Lactobacillus acidophilus*, and *Bifidobacterium bifidum*) QD via enteral tube for the period of MV (less than 28 days)	Placebo	Chest radiograph, purulent tracheal secretions, fever, leukocytosis, and positive quantitative cultures of distal pulmonary secretions
Morrow et al. (27)	Double blinded, single-center, in the USA	138	Critically ill adult patients	2 × 10^9^ CFU of *Lactobacillus rhamnosus* BID via nasogastric tube until extubation	Placebo	New and persistent infiltrate on chest radiographs with two of three supporting findings: fever, leukocytosis, and purulent sputum
Knight et al. (28)	Double blinded, single-center, in the UK	259	Critically ill adult patients	1 × 10^10^ of *Pediococcus pentosaceus, Leuconostoc mesenteroides, Lactobacillus paracasei subsp paracasei* and *Lactiplantibacillus paraplantarum* BID via nasogastric or orogastric tube with 24 h of ICU admission, until 28 day after admission	Placebo	Pneumonia occurring more than 48 h after endotracheal intubation
Giamarellos-Bourboulis et al. (29)	Double blinded, multicenter, in Greece	72	Multiple injured patients	Synbiotic preparation consisted of a combination of 10^11^ CFU of each of four probiotics; *Pediococcus pentoseceus, Leuconostoc mesenteroides, Lactobacillus paracasei*, and *Lactiplantibacillus paraplantarum* per day for a 15-day study period	Placebo	New or persistent consolidation in lung X-ray, purulent TBS, and clinical pulmonary infection score
Forestier et al. (30)	Double blinded, single-center, in France	208	Critically ill adult patients	10^9^ CFU of *Lacticaseibacillus rhamnosus* BID via nasogastric tube, started at the 3rd day of ICU admission, until ICU discharge or death	Placebo	Positive quantitative sample, abnormal radiographical and progressive parenchymatous infiltrates
Spindler-Vesel et al. (31)	Double blinded, single-center, in Slovenia	55	Multiple injured patients	Synbiotic consisting of 10^10^ *Pediococcus pentosaceus*, 10^10^ *Ligilactobacillus araffinosus*, 10^10^ *Lactobacillus paracasei subsp paracasei*, 10^10^ *Lactiplantibacillus paraplantarum*, the study period was 7 days	No placebo	Microbiological specimens

### Statistical Synthesis and Analysis

Dichotomous data were presented as odds ratio (*OR*) with 95% *CI*; continuous data were presented as mean difference (*MD*) with 95% *CI* for heterogeneity between studies were tested by the Chi-squared test with significance set at a *p*-value of 0.1, and quantitatively by inconsistency (*I*^2^) statistics (33). Significant heterogeneity was suggested when *I*^2^ value >50% and a random-effect model was used. In addition, the funnel plot and Egger's regression test were employed to investigate potential publication bias.

For the incidence of VAP, a predefined subgroup analysis was performed by the definition of VAP (quantitative microbiological confirmed VAP vs. non-quantitative microbiological confirmed VAP). The number needed to treat (NNT) analysis (34) was performed for the incidence of VAP, mortality, diarrhea, and bacterial colonization to provide an understanding of the incremental benefits of administering probiotics relative to usual care. Furthermore, a sensitivity analysis was employed to examine the effect of individual studies by omitting each one at a time.

## Results

### Study Characteristics

**Figure 1:**
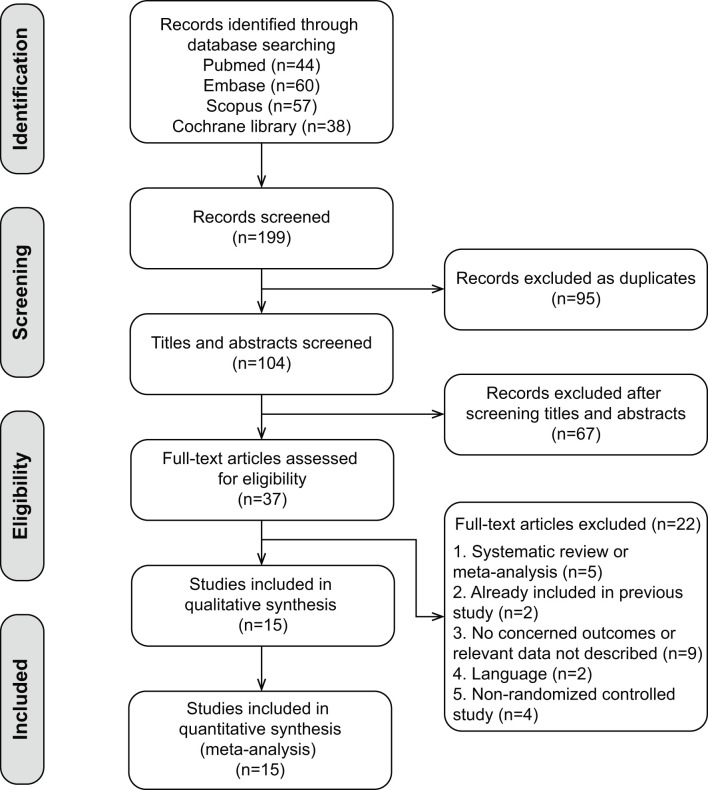
Flowchart of selection for the meta-analysis.

A total of 199 studies were initially retrieved from the above databases, such as 44 from Pubmed, 60 from Embase, 57 from Scopus, and 38 from Cochrane library. Ninety-five were excluded due to duplicates, the remaining 104 articles were screened based upon the review of the titles and abstracts, 67 studies were excluded. Full texts of the remaining 37 articles were independently assessed by two investigators to determine inclusion and exclusion. After strict screening according to inclusion and exclusion criteria, 22 studies were excluded, 15 RCTs (16, 18–31) were finally included in our meta-analysis (Figure 1 shows the study selection).

Characteristics of included trials are shown in Table 1. These trials were published between 2007 and 2021. The sample sizes of included trials were ranged from 52 to 2,650 (the total number was 4,693). Most of the studies enrolled a mixed population of critically ill adult patients, three studies (25, 29, 31) included patients with severe trauma, Shimizu et al. (20) focused on patients with sepsis and Banupriya et al. (22) performed their trial in a pediatric intensive care unit. Furthermore, according to the new taxonomic description (35), the species and strain of probiotics administration varied in these studies. In six of the studies (16, 19, 23, 24, 27, 30), a single probiotic was used, such as *Lacticaseibacillus rhamnosus* (35) in Johnstone et al. (16), Morrow et al. (27), and Forestier et al. (30), *Lactiplantibacillus paraplantarum* (35) in Klarin et al. (19) and Oudhuis et al. (24), *Lactobacillus casei* (35) in Rongrungruang et al. (23). The rest of the nine studies used multiple probiotics. Furthermore, only one study (19) administrated probiotics as oral care, whereas others supplemented probiotics through the enteral route. Of the 15 included studies, all the studies reported the incidence of VAP. The incidence of VAP ranged from 7 to 81%, with an average incidence of 24%. In addition, the definition of VAP was varied among all studies, six studies (18, 23–26, 30) used the quantitative microbiological test to define VAP, the rest of included studies used non-quantitative microbiological or clinical features to define VAP.

### Quality Assessment

**Figure 2:**
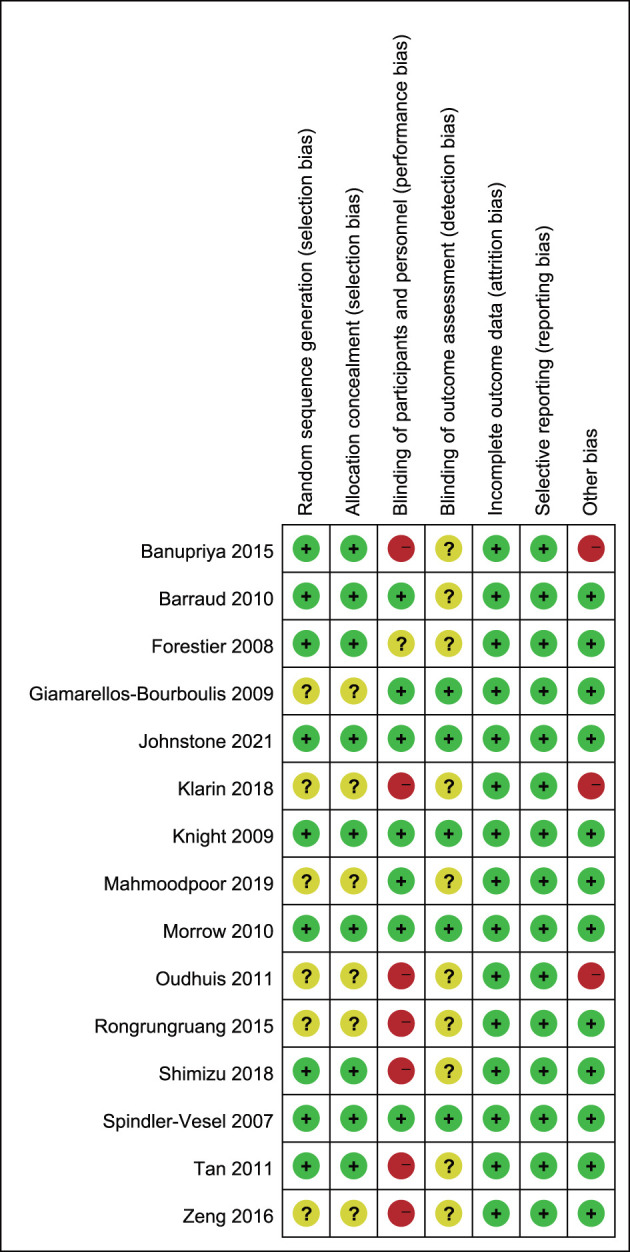
Risk of bias summary.

The risk of bias assessment was summarized in Figure 2. Six studies did not report the details of random sequence generation and allocation concealment. Seven studies were rated as high risk of bias since five studies (19, 21–24) were open-label trials and two (20, 25) were single-blind trials. Moreover, the blinding method for outcome assessment was not reported in ten studies, which would either underestimate or overestimate the size of the effect. Furthermore, three studies had other biases: Klarin et al. (19) only administrated probiotics as oral care, patients in the control group received selective decontamination of the digestive tract in the trial by Oudhuis et al. (24). Banupriya et al. (22) focused on critically ill children in the pediatric intensive care unit.

The test of asymmetry on the funnel plot and Egger's test was concluded for every outcome (Supplementary Material 3). Potential publication bias was observed for the incidence of VAP and diarrhea (Egger's test: *p* < 0.10), thus, an analysis using the trim and fill method was performed. After imputing, the funnel plot became symmetrical and the pooled estimate showed no association between probiotics supplementation and the incidence of VAP (*OR* 0.92, 95% *CI* 0.64 to 1.33) or diarrhea (*OR* 1.08, 95% *CI* 0.80 to 1.46).

### Primary Outcome

**Figure 3:**
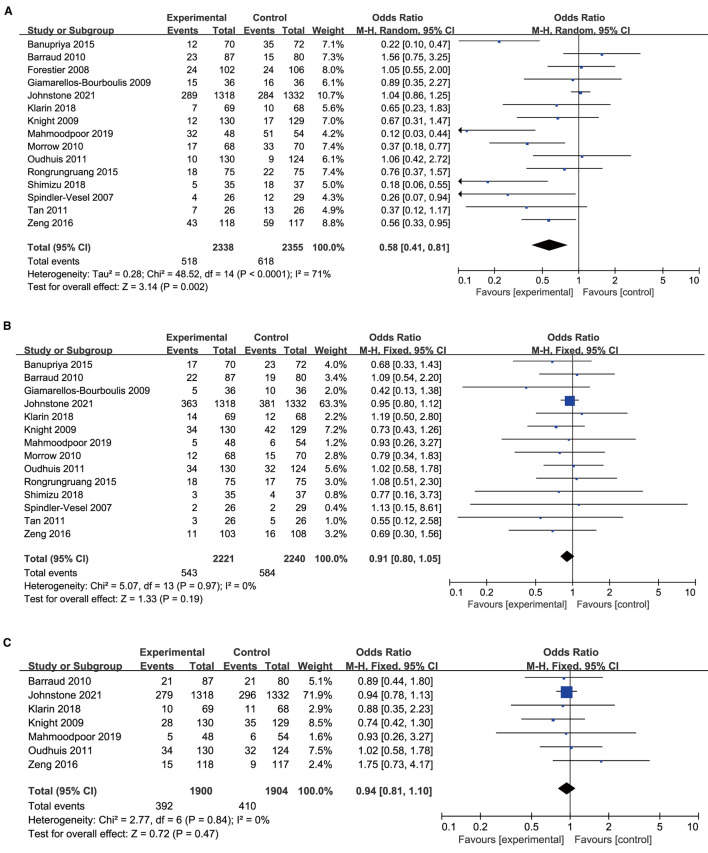
Forest plots for the effect of probiotics on **(A)** incidence of VAP; **(B)** overall mortality; **(C)** ICU mortality.

All included studies with 4,693 participants, 2,338 in the probiotics group, and 2,355 in the placebo group reported the incidence of VAP. The analysis showed that the incidence of VAP in the probiotic group was significantly lower than that in the control group (*OR* 0.58, 95% *CI* 0.41 to 0.81; *p* = 0.002; *I*^2^ = 71%; Figure 3A). High heterogeneity was seen between studies. The NNT to prevent one patient of VAP in mechanical ventilated patients in ICU was 24.

A total of 14 trials with 4,461 patients were included in the meta-analysis examining the association between mortality and probiotic intervention. The studies were found to be homogenous, there was no significant association between probiotics and mortality (*OR* 0.91, 95% *CI* 0.80 to 1.05; *p* = 0.19; *I*^2^ = 0%; Figure 3B). The NNT to prevent one patient of death in mechanical ventilated patients in ICU was 62. Furthermore, there was no significant difference in ICU mortality in the probiotic group vs. the control group (*OR* 0.94, 95% *CI* 0.81 to 1.10; *p* = 0.47; *I*^2^ = 0%; Figure 3C).

### Secondary Outcome

**Figure 4:**
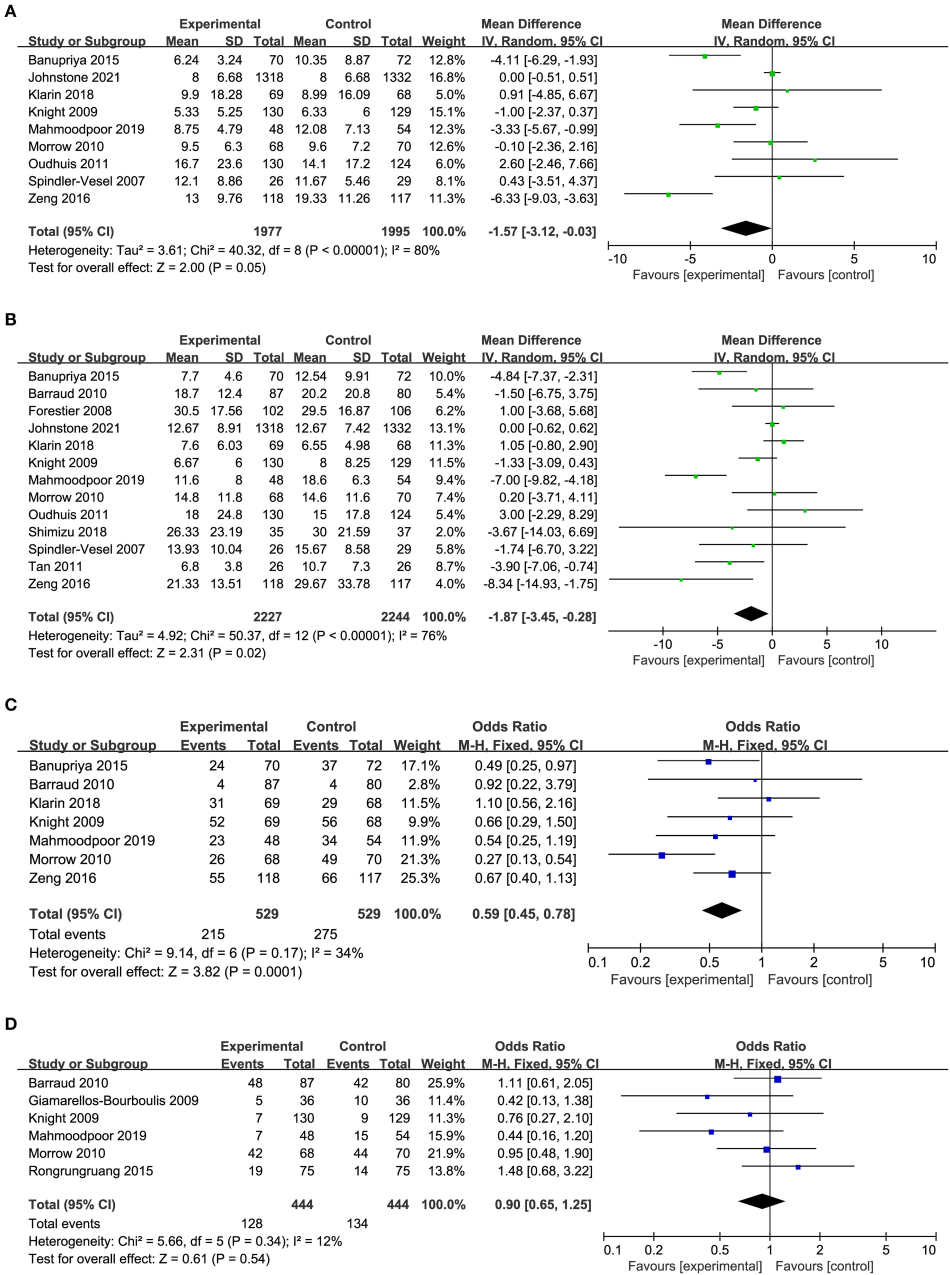
Forest plots for the effect of probiotics on **(A)** duration of MV; **(B)** length of ICU stay; **(C)** bacteria colonization; **(D)** incidence of diarrhea.

Nine studies including 1,977 patients in the probiotics group and 1,995 patients in the control group provided data on the duration of mechanical ventilation. The duration of mechanical ventilation in the probiotics group was shorter than the control group, there was high heterogeneity between the two groups (*MD* −1.57, 95% *CI* −3.12 to −0.03; *p* = 0.05; *I*^2^ = 80%; Figure 4A).

A total of 13 studies with 909 in the probiotics group and 912 in the control group reported the length of ICU stay. Pooled data demonstrated that the length of ICU stay of patients in the probiotics group was shorter than that in the control group, there was high heterogeneity between the two groups (*MD* −1.87, 95% *CI* −3.45 to −0.28; *p* = 0.02; *I*^2^ = 76%; Figure 4B).

Seven of the 14 studies included 529 patients in probiotics and 529 patients in the control group were pooled to analyze the bacterial colonization, a significant reduction was found in the probiotics group than the control group (*OR* 0.59, 95% CI 0.45 to 0.78; *p* = 0.0001; *I*^2^ = 34%; Figure 4C), and a moderate heterogeneity was seen between the studies. The NNT to prevent one patient of bacterial colonization in mechanical ventilated patients in ICU was 9.

Six studies including 888 patients with 444 in the probiotics group and 444 in the control group reported on diarrhea, no statistically significant differences were observed regarding diarrhea between the probiotics group and control group, a low heterogeneity was seen between the studies (*OR* 0.90, 95% *CI* 0.65 to 1.25; *p* = 0.54; *I*^2^ = 12%; Figure 4D). The NNT to prevent one patient of diarrhea in mechanical ventilated patients in ICU was 74.

### Subgroup and Sensitivity Analyses

**Figure 5:**
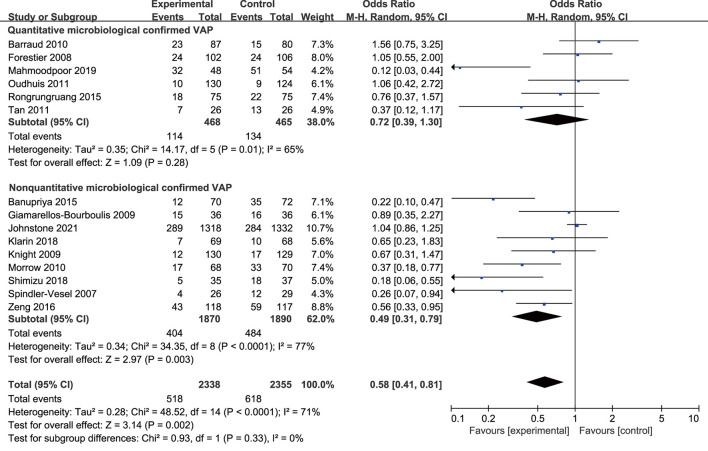
Forest plots for the subgroup analysis on VAP: quantitative microbiological confirmed VAP vs. non-quantitative microbiological confirmed VAP.

The subgroup analysis of quantitative microbiological confirmed VAP (*OR* 0.72, 95% *CI* 0.39 to 1.30; *p* = 0.28; *I*^2^ = 65%) showed no significant association between probiotics and VAP. However, the subgroup of non-quantitative microbiological confirmed VAP (*OR* 0.49, 95% *CI* 0.31 to 0.79; *p* = 0.003; *I*^2^ = 77%) suggested a reduction of VAP incidence. The difference between the subgroups was not statistically significant (*p* = 0.33; Figure 5).

**Figure 6:**
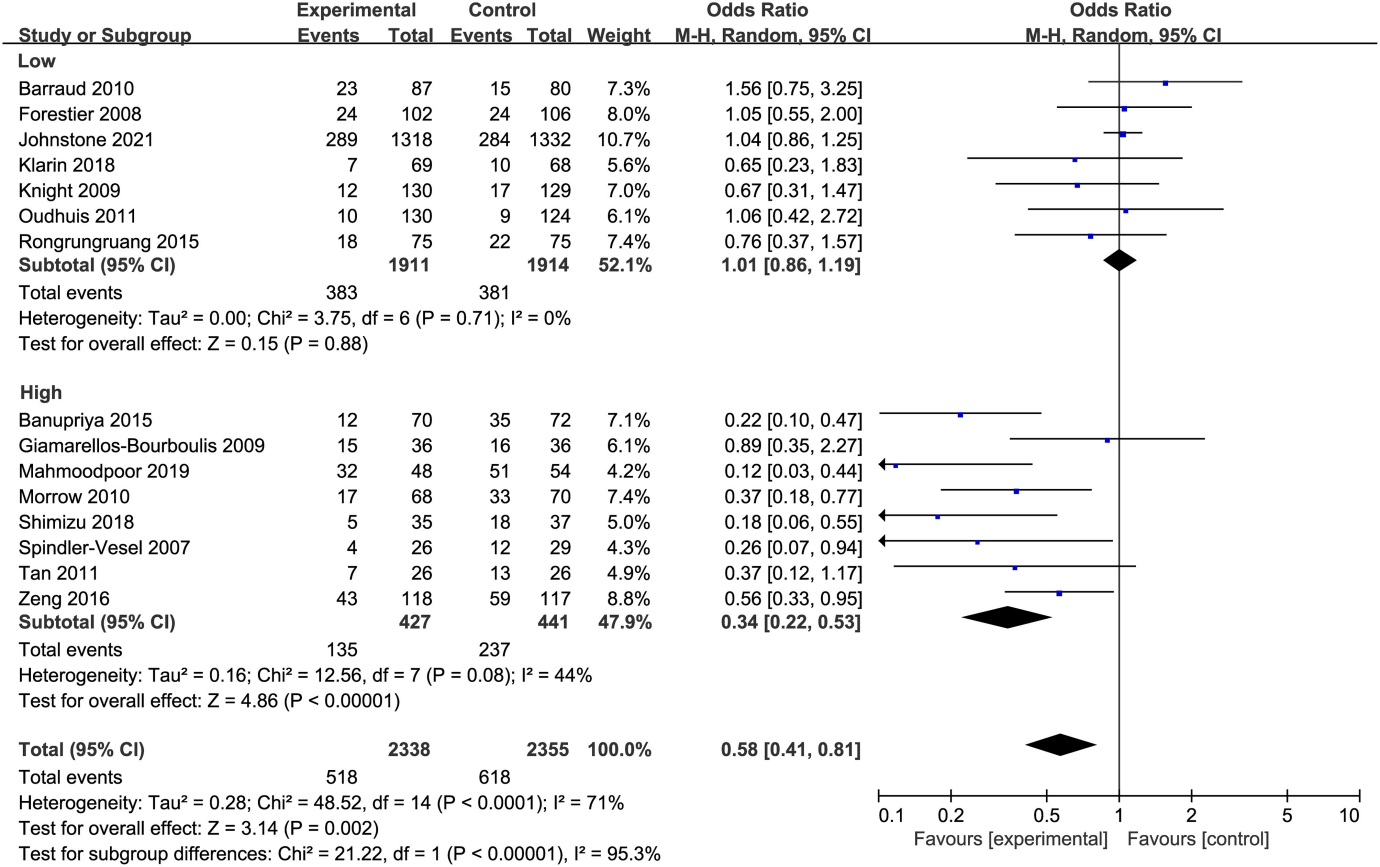
Forest plots for the subgroup analysis on VAP: low vs. high incidence of VAP.

**Figure 7:**
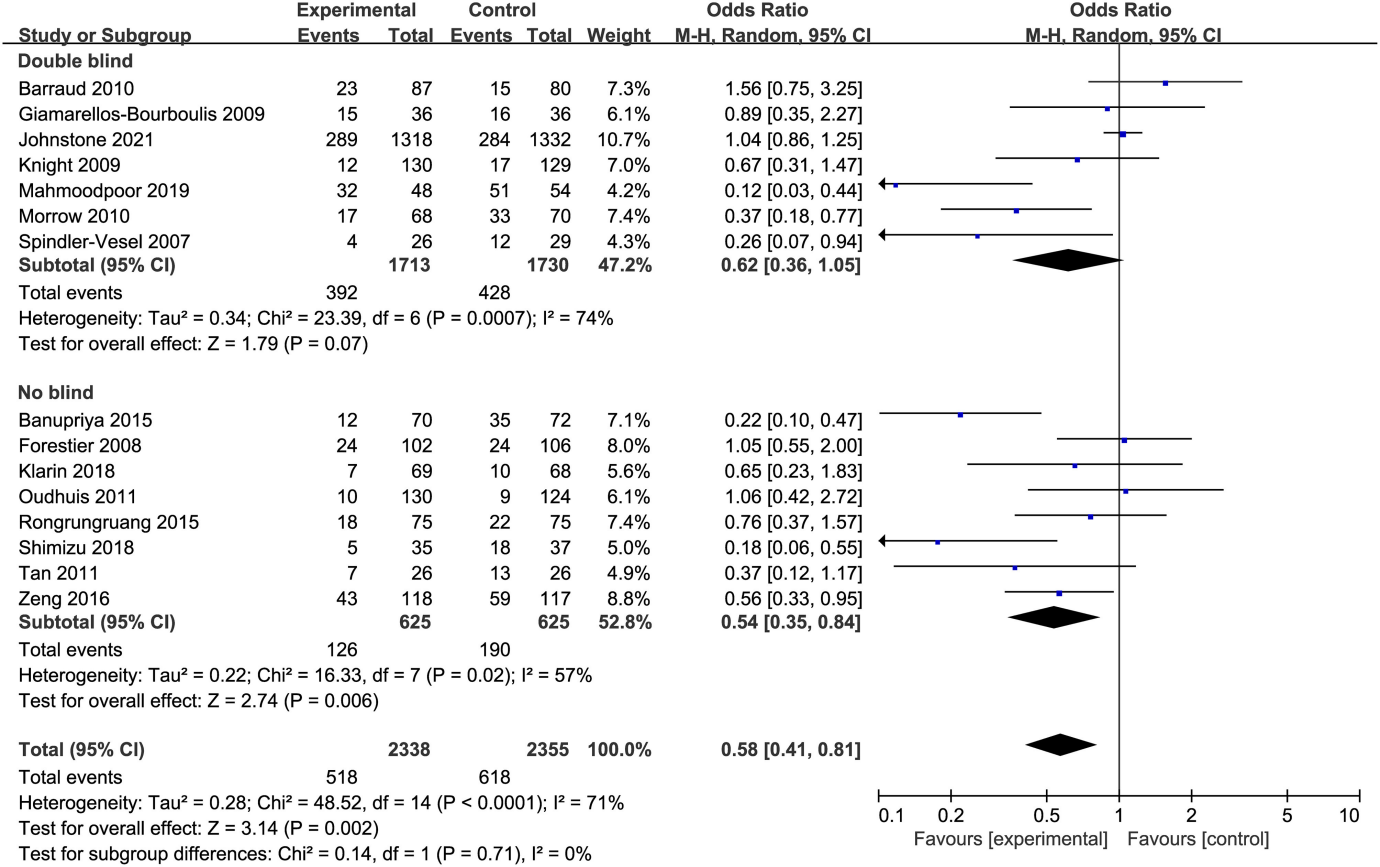
Forest plots for the subgroup analysis on VAP: double blind RCTs vs. no blind RCTs.

Moreover, according to the average incidence of VAP, patients were divided into the low and high incidence of VAP groups. The effect of probiotics in reducing the incidence of VAP was statistically significant in trials with high incidence of VAP (*OR* 0.34, 95% *CI* 0.22 to 0.53; *p* < 0.001; *I*^2^ = 44%) while it was not significant in those with relatively low incidence of VAP (*OR* 1.01, 95% *CI* 0.86 to 1.19; *p* = 0.88; *I*^2^ = 0%; Figure 6). In addition, since only six studies were double-blind trials, subgroup analyses were performed based on the differences in trial design. The subgroup analysis of the double-blind studies showed no effect of probiotics compared with controls on the prevention of VAP (*OR* 0.62, 95% *CI* 0.36 to 1.05; *p* = 0.07; *I*^2^ = 74%; Figure 7), while the subgroup of no-blind studies still showed a protective effect (*OR* 0.54, 95% *CI* 0.35 to 0.84; *p* = 0.006; *I*^2^ = 57%; Figure 7).

Since the clinical features of included population, types and dosage of probiotics, and definition of VAP were not constant among included studies, a sensitivity analysis was performed by removing each trial to examine the effect of individual study on the overall result. The sensitivity analysis showed similar results to the overall analysis, indicating good robustness (Supplementary Material 4).

## Discussion

Our meta-analysis included a total of 15 trials with 4,693 participants to evaluate the effect of probiotics in the prevention of VAP. As far as we know, this is the largest and most updated meta-analysis to evaluate the effects of probiotics in critically ill patients. The analysis of the outcomes demonstrated that probiotics significantly decreased the incidence of VAP, and the NNT to prevent one patient of VAP in mechanical ventilated patients in ICU was 24. Moreover, the use of probiotics in mechanical ventilated patients was associated with reduced duration of mechanical ventilation, length of ICU stay, and bacteria colonization. However, no appreciable effects were conferred by probiotics on ICU mortality and the occurrence of diarrhea. Moreover, subgroup analysis showed that the definition of VAP was a potential source of heterogeneity. The probiotic had no significant effect on the quantitative microbiological confirmed VAP, while the protective effect was proved by other RCTs which used the non-quantitative microbiological method to define VAP. Thus, to further evaluate the effect of probiotics in VAP, large scale multicentric studies with a unified definition of VAP are needed in the future. In addition, in patients with a high risk of developing VAP, the beneficial effect of probiotics in reducing the incidence of VAP was more significant, while there was no significant difference in patients with a low incidence of VAP.

Our results are generally consistent with the recent meta-analyses (10–13) that the use of probiotics was associated with a significant reduction in VAP but no significant difference in overall mortality. However, our results demonstrated that the use of probiotics could reduce the length of ICU stay, duration of mechanical ventilation, and incidence of bacteria colonization. Compared with previous meta-analyses by Zhao et al. (13) and Ji et al. (12), the trials by Klarin et al. (36) and Kotzampassi et al. (37) were excluded in our meta-analysis because the patient cohorts of these two trials are the same as in Klarin et al. (19) and Giamarellos-Bourboulis et al. (29). Furthermore, our meta-analysis included the latest study by Johnstone et al. (16), which enrolled more than 2,600 patients and was the largest randomized trial to date. In addition, our meta-analysis also found the beneficial effect of probiotics in reducing the incidence of VAP was more effective in patients at high risk of developing into VAP. It is a common complication in patients receiving invasive mechanical ventilation, identifying effective preventive measures of VAP is difficult because of the many factors involved and the lack of a unique definition. Even though two meta-analyses did not find a statistically significant decrease in the incidence of VAP (38, 39), more studies include our owns have shown the decrease in the incidence of VAP after probiotic administration (10, 40, 41). The different effects may depend on the patient population and the probiotic strain studied. Many observations suggest that probiotic therapy is benefit for administration in a properly selected, critically ill population admitted to the surgical ICU (18, 42). In contrast, studies suggested that probiotics should not be administered in immunosuppressed patients or patients with severe acute pancreatitis (15, 40).

The pathogenesis of VAP is mainly due to bacterial colonization of the upper respiratory tract and inhalation of contaminated secretions into the lower respiratory tract. Our results demonstrated that compared with the control group, the probiotics group decreases bacterial colonization. The positive effects of probiotics on VAP may include (1) inducing host cell antimicrobial peptides and strengthening the gut barrier function, (2) reducing the overgrowth of potential pathogens and anti-oxidative activity, and (3) stimulating immune responses and mucus and IgA production (43). Gut flora plays a central role in the maintenance of the gut barrier and a healthy gut microbiome, probiotic therapy may be essential to optimizing outcomes in critically ill patients (44). It is worth noting that for prevention of VAP in clinical, bundles that combine multiple prevention strategies are associated with earlier extubation and lower mortality rates, such as avoiding intubation and exposure to invasive mechanical ventilation by using high flow oxygen or non-invasive ventilation, lightening sedation, using spontaneous breathing trials to prompt early extubation, and early mobilization may be more effective (45).

In numerous clinical trials, probiotics are associated with a reduction in antibiotic-associated diarrhea (46, 47). In our study, although there was a reduction in VAP incidence, duration of mechanical ventilation, and length of ICU stay, probiotic therapy did not decrease other clinical endpoints such as ICU mortality and the occurrence of diarrhea, which are consistent with the previous meta-analysis (10). Antibiotic-associated diarrhea is characterized by the disruption of the gut microbiota altering water absorption and ultimately inducing diarrhea. One of the uses of probiotics is for the prevention of antibiotic-associated diarrhea (48). However, none of the included trials reported detailed information on antibiotic use between intervention and control groups. Thus, whether probiotics alone do not cause diarrhea or probiotics do not improve antibiotic-associated diarrhea in VAP requires a more definite subgroup analysis.

The strength of this meta-analysis includes the use of standard methods to reduce bias (comprehensive literature search, duplicate data abstraction, specific criteria for searching and analysis), and the analysis of relevant clinical outcomes in the critically ill. Additional conduct of explicit subgroup and sensitivity analysis provides evidence in the robustness of estimates.

However, there are some limitations of this meta-analysis. First, the main study limitation is the limited number of included studies and small sample size. Due to the restriction of the retrieval strategy, there was an insufficient sample to strengthen this result. Almost all of the included studies had a sample size of <100 patients, which are typically characterized as small studies. As a result, our study may be subjected to small study effect bias (49). Second, the methodological quality of the included studies was low, with several RCTs clearly lacking illustrations of randomness, allocation concealment, and blinding, among other factors, which increased difficulty in the risk of bias assessment. The subgroup analyses based on the differences in trial design indicated that double-blind studies showed no effect of probiotics compared with controls on the prevention of VAP, which meant that the role of probiotics may be inflated by studies with flawed designs. Third, the diagnosis of VAP was based on various definitions in the included studies, which was a potential source of heterogeneity and may have contributed to inconsistency. In addition, it is worth noting that although no adverse effects related to probiotics were found in the included studies, it is vital to conduct safety monitoring in future clinical trials.

## Conclusion

In conclusion, this comprehensive and updated meta-analysis analyzed the effects of probiotics in mechanical ventilated patients and found that probiotics can reduce the incidence of VAP, duration of mechanical ventilation, length of ICU stay, and bacteria colonization, but there was no significant effect on the mortality and occurrence of diarrhea. However, the significant heterogeneity and publication bias may reduce the credibility of the results. The benefit of probiotics seems clinically plausible but needed more well-designed RCTs to further validate the effect of probiotics for mechanical ventilated patients. Moreover, some new guidelines such as the updated PRISMA checklist 2020 (50), and new tools such as hierarchical nested design or competing event analyses have been recently proposed to improve the design and the analysis of future studies.

## Data Availability Statement

The original contributions presented in the study are included in the article/Supplementary Material, further inquiries can be directed to the corresponding author/s.

## Author Contributions

HS conceived the idea, performed the analysis, and drafted the manuscript. WH and XZ contributed to the study design, data acquisition, and interpretation. JT, SZ, and XS helped in the statistical analysis. WW helped to frame the idea of the study and provided technical support. All the authors read and approved the final manuscript.

## Conflict of Interest

The authors declare that the research was conducted in the absence of any commercial or financial relationships that could be construed as a potential conflict of interest.

## Publisher's Note

All claims expressed in this article are solely those of the authors and do not necessarily represent those of their affiliated organizations, or those of the publisher, the editors and the reviewers. Any product that may be evaluated in this article, or claim that may be made by its manufacturer, is not guaranteed or endorsed by the publisher.

## References

[1] Horan TC Andrus M Dudeck MA. CDC/NHSN surveillance definition of health care-associated infection and criteria for specific types of infections in the acute care setting. Am J Infect Control. (2008) 36:309–32. 10.1016/j.ajic.2008.03.002 18538699

[2] Papazian L Klompas M Luyt CE. Ventilator-associated pneumonia in adults: a narrative review. Intensive Care Med. (2020) 46:888–906. 10.1007/s00134-020-05980-0 32157357 PMC7095206

[3] Safdar N Dezfulian C Collard HR Saint S. Clinical and economic consequences of ventilator-associated pneumonia: a systematic review. Crit Care Med. (2005) 33:2184–93. 10.1097/01.CCM.0000181731.53912.D9 16215368

[4] Melsen WG Rovers MM Groenwold RH Bergmans DC Camus C Bauer TT . Attributable mortality of ventilator-associated pneumonia: a meta-analysis of individual patient data from randomised prevention studies. Lancet Infect Dis. (2013) 13:665–71. 10.1016/S1473-3099(13)70081-1 23622939

[5] Muscedere J Sinuff T Heyland DK Dodek PM Keenan SP Wood G . The clinical impact and preventability of ventilator-associated conditions in critically ill patients who are mechanically ventilated. Chest. (2013) 144:1453–60. 10.1378/chest.13-0853 24030318

[6] Schreiber MP Shorr AF. Challenges and opportunities in the treatment of ventilator-associated pneumonia. Expert Rev Anti Infect Ther. (2017) 15:23–32. 10.1080/14787210.2017.1250625 27771978

[7] Hill C Guarner F Reid G Gibson GR Merenstein DJ Pot B . Expert consensus document. The International Scientific Association for Probiotics and Prebiotics consensus statement on the scope and appropriate use of the term probiotic. Nat Rev Gastroenterol Hepatol. (2014) 11:506–14. 10.1038/nrgastro.2014.66 24912386

[8] Vincent JL de Souza Barros D Cianferoni S. Diagnosis, management and prevention of ventilator-associated pneumonia: an update. Drugs. (2010) 70:1927–44. 10.2165/11538080-000000000-00000 20883051

[9] Ghosh S van Heel D Playford RJ. Probiotics in inflammatory bowel disease: is it all gut flora modulation? Gut. (2004) 53:620–2. 10.1136/gut.2003.034249 15082574 PMC1774052

[10] Batra P Soni KD Mathur P. Efficacy of probiotics in the prevention of VAP in critically ill ICU patients: an updated systematic review and meta-analysis of randomized control trials. J Intensive Care. (2020) 8:81. 10.1186/s40560-020-00487-8 33082958 PMC7561245

[11] Su M Jia Y Li Y Zhou D Jia J. Probiotics for the prevention of ventilator-associated pneumonia: a meta-analysis of randomized controlled trials. Respir Care. (2020) 65:673–85. 10.4187/respcare.07097 32127415

[12] Ji T Zhu X Shang F Zhang X. Preventive effect of probiotics on ventilator-associated pneumonia: a meta-analysis of 2428 patients. Ann Pharmacother. (2021) 55:949–62. 10.1177/1060028020983021 33349001

[13] Zhao J Li LQ Chen CY Zhang GS Cui W Tian BP. Do probiotics help prevent ventilator-associated pneumonia in critically ill patients? A systematic review with meta-analysis. ERJ Open Res. (2021) 7:00302. 10.1183/23120541.00302-2020 33532460 PMC7836470

[14] Sherid M Samo S Sulaiman S Husein H Sifuentes H Sridhar S. Liver abscess and bacteremia caused by lactobacillus: role of probiotics? Case report and review of the literature. BMC Gastroenterol. (2016) 16:138. 10.1186/s12876-016-0552-y 27863462 PMC5116133

[15] Doron S Snydman DR. Risk and safety of probiotics. Clin Infect Dis. (2015) 60:S129–134. 10.1093/cid/civ085 25922398 PMC4490230

[16] Johnstone J Meade M Lauzier F Marshall J Duan E Dionne J . Effect of probiotics on incident ventilator-associated pneumonia in critically ill patients: a randomized clinical trial. JAMA. (2021) 326:1024–33. 10.1001/jama.2021.13355 34546300 PMC8456390

[17] Liberati A Altman DG Tetzlaff J Mulrow C Gøtzsche PC Ioannidis JP . The PRISMA statement for reporting systematic reviews and meta-analyses of studies that evaluate healthcare interventions: explanation and elaboration. BMJ. (2009) 339:b2700. 10.1136/bmj.b2700 19622552 PMC2714672

[18] Mahmoodpoor A Hamishehkar H Asghari R Abri R Shadvar K Sanaie S. Effect of a probiotic preparation on ventilator-associated pneumonia in critically ill patients admitted to the intensive care unit: a prospective double-blind randomized controlled trial. Nutr Clin Pract. (2019) 34:156–62. 10.1002/ncp.10191 30088841

[19] Klarin B Adolfsson A Torstensson A Larsson A. Can probiotics be an alternative to chlorhexidine for oral care in the mechanically ventilated patient? A multicentre, prospective, randomised controlled open trial. Crit Care. (2018) 22:272. 10.1186/s13054-018-2209-4 30368249 PMC6204275

[20] Shimizu K Yamada T Ogura H Mohri T Kiguchi T Fujimi S . Synbiotics modulate gut microbiota and reduce enteritis and ventilator-associated pneumonia in patients with sepsis: a randomized controlled trial. Crit Care. (2018) 22:239. 10.1186/s13054-018-2167-x 30261905 PMC6161427

[21] Zeng J Wang CT Zhang FS Qi F Wang SF Ma S Wu TJ . Effect of probiotics on the incidence of ventilator-associated pneumonia in critically ill patients: a randomized controlled multicenter trial. Intensive Care Med. (2016) 42:1018–28. 10.1007/s00134-016-4303-x 27043237

[22] Banupriya B Biswal N Srinivasaraghavan R Narayanan P Mandal J. Probiotic prophylaxis to prevent ventilator associated pneumonia (VAP) in children on mechanical ventilation: an open-label randomized controlled trial. Intensive Care Med. (2015) 41:677–85. 10.1007/s00134-015-3694-4 25708419

[23] Rongrungruang Y Krajangwittaya D Pholtawornkulchai K Tiengrim S Thamlikitkul V. Randomized controlled study of probiotics containing Lactobacillus casei (Shirota strain) for prevention of ventilator-associated pneumonia. J Med Assoc Thai. (2015) 98:253–9. 25920295

[24] Oudhuis GJ Bergmans DC Dormans T Zwaveling JH Kessels A Prins MH . Probiotics versus antibiotic decontamination of the digestive tract: infection and mortality. Intensive Care Med. (2011) 37:110–7. 10.1007/s00134-010-2002-6 20721536 PMC3020315

[25] Tan M Zhu JC Du J Zhang LM Yin HH. Effects of probiotics on serum levels of Th1/Th2 cytokine and clinical outcomes in severe traumatic brain-injured patients: a prospective randomized pilot study. Crit Care. (2011) 15:R290. 10.1186/cc10579 22136422 PMC3388628

[26] Barraud D Blard C Hein F Marcon O Cravoisy A Nace L . Probiotics in the critically ill patient: a double blind, randomized, placebo-controlled trial. Intensive Care Med. (2010) 36:1540–7. 10.1007/s00134-010-1927-0 20502866

[27] Morrow LE Kollef MH Casale TB. Probiotic prophylaxis of ventilator-associated pneumonia: a blinded, randomized, controlled trial. Am J Respir Crit Care Med. (2010) 182:1058–64. 10.1164/rccm.200912-1853OC 20522788 PMC2970846

[28] Knight DJ Gardiner D Banks A Snape SE Weston VC Bengmark S . Effect of synbiotic therapy on the incidence of ventilator associated pneumonia in critically ill patients: a randomised, double-blind, placebo-controlled trial. Intensive Care Med. (2009) 35:854–61. 10.1007/s00134-008-1368-1 19083199

[29] Giamarellos-Bourboulis EJ Bengmark S Kanellakopoulou K Kotzampassi K. Pro- and synbiotics to control inflammation and infection in patients with multiple injuries. J Trauma. (2009) 67:815–21. 10.1097/TA.0b013e31819d979e 19820590

[30] Forestier C Guelon D Cluytens V Gillart T Sirot J De Champs C. Oral probiotic and prevention of Pseudomonas aeruginosa infections: a randomized, double-blind, placebo-controlled pilot study in intensive care unit patients. Crit Care. (2008) 12:R69. 10.1186/cc6907 18489775 PMC2481460

[31] Spindler-Vesel A Bengmark S Vovk I Cerovic O Kompan L. Synbiotics, prebiotics, glutamine, or peptide in early enteral nutrition: a randomized study in trauma patients. JPEN J Parenter Enteral Nutr. (2007) 31:119–26. 10.1177/0148607107031002119 17308252

[32] Higgins JP Altman DG Gøtzsche PC Jüni P Moher D Oxman AD . The Cochrane Collaboration's tool for assessing risk of bias in randomised trials. BMJ. (2011) 343:d5928. 10.1136/bmj.d5928 22008217 PMC3196245

[33] Higgins JP Thompson SG Deeks JJ Altman DG. Measuring inconsistency in meta-analyses. BMJ. (2003) 327:557–60. 10.1136/bmj.327.7414.557 12958120 PMC192859

[34] Cook RJ Sackett DL. The number needed to treat: a clinically useful measure of treatment effect. BMJ. (1995) 310:452–4. 10.1136/bmj.310.6977.452 7873954 PMC2548824

[35] Zheng J Wittouck S Salvetti E Franz C Harris HMB Mattarelli P . A taxonomic note on the genus Lactobacillus: Description of 23 novel genera, emended description of the genus Lactobacillus Beijerinck 1901, and union of Lactobacillaceae and Leuconostocaceae. Int J Syst Evol Microbiol. (2020) 70:2782–858. 10.1099/ijsem.0.004107 32293557

[36] Klarin B Molin G Jeppsson B Larsson A. Use of the probiotic Lactobacillus plantarum 299 to reduce pathogenic bacteria in the oropharynx of intubated patients: a randomised controlled open pilot study. Crit Care. (2008) 12:R136. 10.1186/cc7109 18990201 PMC2646346

[37] Kotzampassi K Giamarellos-Bourboulis EJ Voudouris A Kazamias P Eleftheriadis E. Benefits of a synbiotic formula (Synbiotic 2000Forte) in critically Ill trauma patients: early results of a randomized controlled trial. World J Surg. (2006) 30:1848–55. 10.1007/s00268-005-0653-1 16983476

[38] Gu WJ Wei CY Yin RX. Lack of efficacy of probiotics in preventing ventilator-associated pneumonia probiotics for ventilator-associated pneumonia: a systematic review and meta-analysis of randomized controlled trials. Chest. (2012) 142:859–68. 10.1378/chest.12-0679 22797719

[39] Wang J Liu KX Ariani F Tao LL Zhang J Qu JM. Probiotics for preventing ventilator-associated pneumonia: a systematic review and meta-analysis of high-quality randomized controlled trials. PLoS ONE. (2013) 8:e83934. 10.1371/journal.pone.0083934 24367620 PMC3867481

[40] Manzanares W Lemieux M Langlois PL Wischmeyer PE. Probiotic and synbiotic therapy in critical illness: a systematic review and meta-analysis. Crit Care. (2016) 19:262. 10.1186/s13054-016-1434-y 27538711 PMC4991010

[41] Weng H Li JG Mao Z Feng Y Wang CY Ren XQ . Probiotics for preventing ventilator-associated pneumonia in mechanically ventilated patients: a meta-analysis with trial sequential analysis. Front Pharmacol. (2017) 8:717. 10.3389/fphar.2017.00717 29062279 PMC5640711

[42] Sawas T Al Halabi S Hernaez R Carey WD Cho WK. Patients receiving prebiotics and probiotics before liver transplantation develop fewer infections than controls: a systematic review and meta-analysis. Clin Gastroenterol Hepatol. (2015). 13:1567–74.e1563. 10.1016/j.cgh.2015.05.027 26044318

[43] Klingensmith NJ Coopersmith CM. The gut as the motor of multiple organ dysfunction in critical illness. Crit Care Clin. (2016) 32:203–12. 10.1016/j.ccc.2015.11.004 27016162 PMC4808565

[44] Mittal R Coopersmith CM. Redefining the gut as the motor of critical illness. Trends Mol Med. (2014) 20:214–23. 10.1016/j.molmed.2013.08.004 24055446 PMC3959633

[45] Harris BD Thomas GA Greene MH Spires SS Talbot TR. Ventilator bundle compliance and risk of ventilator-associated events. Infect Control Hosp Epidemiol. (2018) 39:637–43. 10.1017/ice.2018.30 29770752

[46] Hempel S Newberry SJ Maher AR Wang Z Miles JN Shanman R . Probiotics for the prevention and treatment of antibiotic-associated diarrhea: a systematic review and meta-analysis. JAMA. (2012) 307:1959–69. 10.1001/jama.2012.3507 22570464

[47] Goldenberg JZ Lytvyn L Steurich J Parkin P Mahant S Johnston BC. Probiotics for the prevention of pediatric antibiotic-associated diarrhea. Cochrane Database Syst Rev. (2015) 12:CD004827. 10.1002/14651858.CD004827.pub4 26695080

[48] Mekonnen SA Merenstein D Fraser CM Marco ML. Molecular mechanisms of probiotic prevention of antibiotic-associated diarrhea. Curr Opin Biotechnol. (2020) 61:226–34. 10.1016/j.copbio.2020.01.005 32087535 PMC7096272

[49] Zhang Z Xu X Ni H. Small studies may overestimate the effect sizes in critical care meta-analyses: a meta-epidemiological study. Crit Care. (2013) 17:R2. 10.1186/cc11919 23302257 PMC4056100

[50] Page MJ McKenzie JE Bossuyt PM Boutron I Hoffmann TC Mulrow CD . The PRISMA 2020 statement: an updated guideline for reporting systematic reviews. BMJ. (2021) 372:n71. 10.1136/bmj.n71 33782057 PMC8005924

