# Supplementary material for: Clinical Benefits From Administering Probiotics to Mechanical Ventilated Patients in Intensive Care Unit: A PRISMA-Guided Meta-Analysis
Source: Front Nutr. 2022 Jan 27;8:798827. doi: 10.3389/fnut.2021.798827 (PMC8829544; doi:10.3389/fnut.2021.798827)
Supplement: Supplementary file 2 [file Table_2.DOCX]

Supplementary Material 2: Searching strategies

Pubmed

#1 probiotic* [Title/Abstract] OR prebiotic* [Title/Abstract] OR symbiotic [Title/Abstract] OR synbiotics [Title/Abstract]

#2 ventilator associated pneumonia [Title/Abstract] OR vap [Title/Abstract]

#3 random*[Title/Abstract] OR randomized control trial [Publication Type]

#1 AND #2 AND #3

Embase

#1 ‘probiotic*’:ab,ti OR ‘prebiotic*’:ab,ti OR ‘symbiotic’:ab,ti OR ‘synbiotics’:ab,ti

#2 (ventilator associated pneumonia):ab,ti OR ‘vap’:ab,ti

#3 (randomized control trial):ab,ti OR ‘random*’:ab,ti

#1 AND #2 AND #3

Scopus

#1 TITLE-ABS-KEY (probiotic*) OR TITLE-ABS-KEY (prebiotic*) OR TITLE-ABS-KEY (symbiotic) OR TITLE-ABS-KEY (synbiotics)

#2 TITLE-ABS-KEY (ventilator associated pneumonia) OR TITLE-ABS-KEY (vap)

#3 TITLE-ABS-KEY (random*) OR TITLE-ABS-KEY (randomized control trial)

#1 AND #2 AND #3

Cochrane Library

#1 ‘probiotic*’:ti,ab,kw OR ‘prebiotic*’: ti,ab,kw OR ‘symbiotic’: ti,ab,kw OR ‘synbiotics’: ti,ab,kw

#2 (ventilator associated pneumonia): ti,ab,kw OR ‘vap’: ti,ab,kw

#3 ‘random*’: ti,ab,kw OR (randomized control trial): ti,ab,kw

#1 AND #2 AND #3
