# Supplementary material for: Clinical Benefits From Administering Probiotics to Mechanical Ventilated Patients in Intensive Care Unit: A PRISMA-Guided Meta-Analysis
Source: Front Nutr. 2022 Jan 27;8:798827. doi: 10.3389/fnut.2021.798827 (PMC8829544; doi:10.3389/fnut.2021.798827)
Supplement: Supplementary file 3 [file Image_1.pdf]

Supplementary Material 3 : Sensitivity analyses

Publication bias assessment by funnel plot and Egger's test

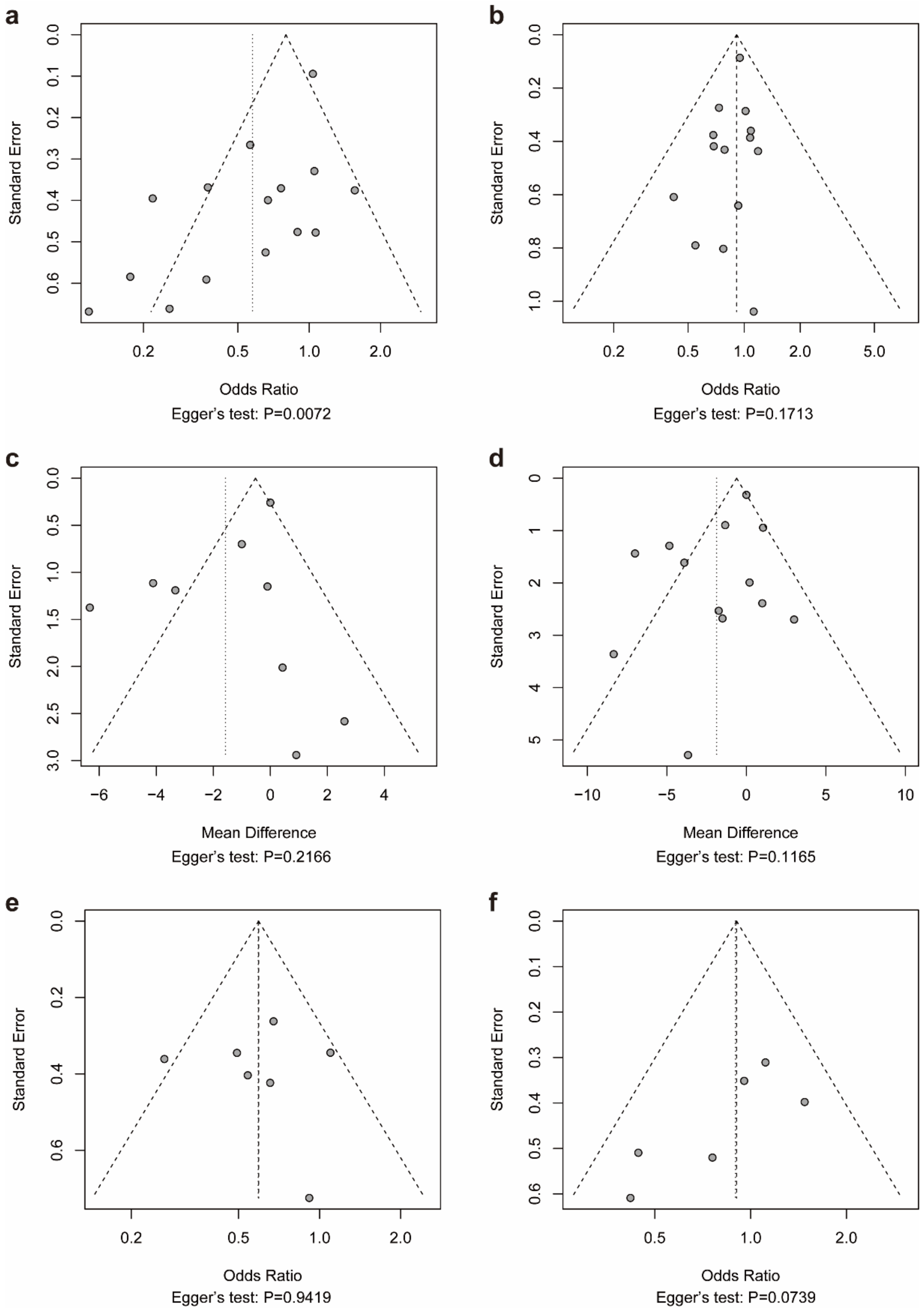

Figure 1: Funnel plot for (a) ventilator associated pneumonia; (b) in-hospital mortality; (c) duration

of mechanical ventilation; (d) length of stay in intensive care unit; (e) incidence of bacterial colonization; (f) incidence of diarrhea

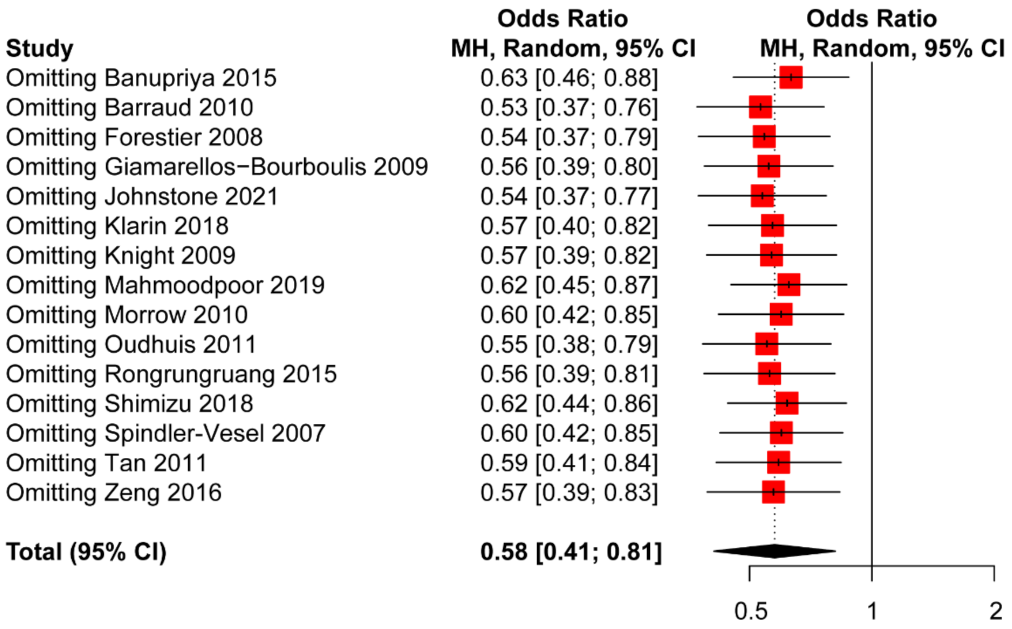

Figure 1: Sensitivity analysis for ventilator associated pneumonia

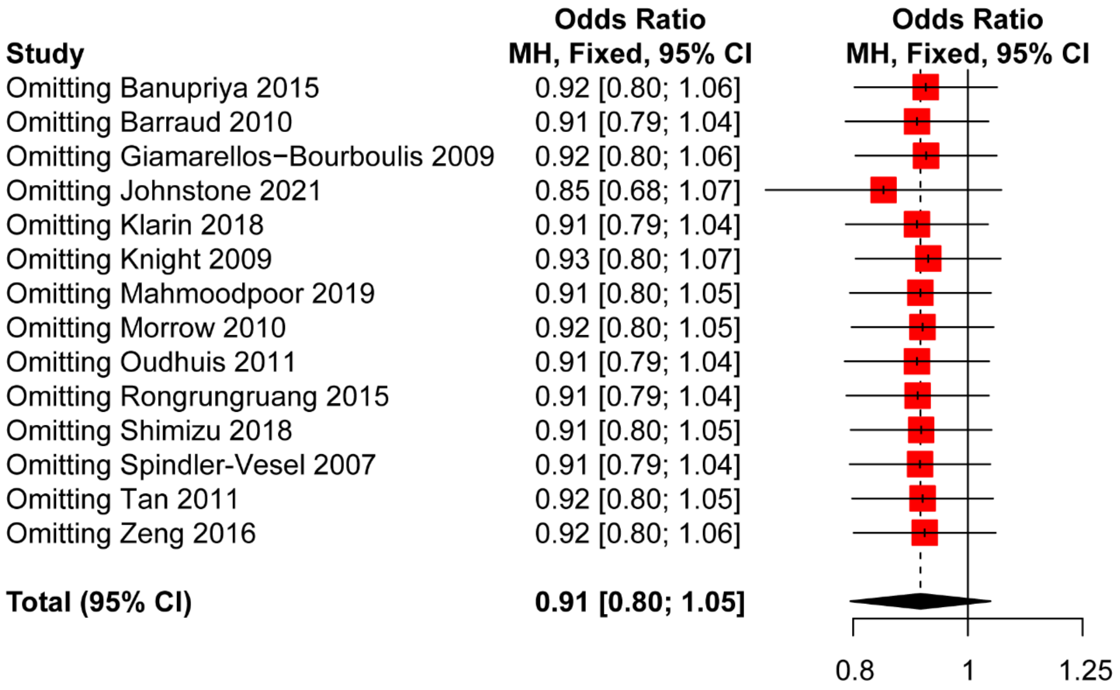

Figure 2: Sensitivity analysis for in-hospital mortality

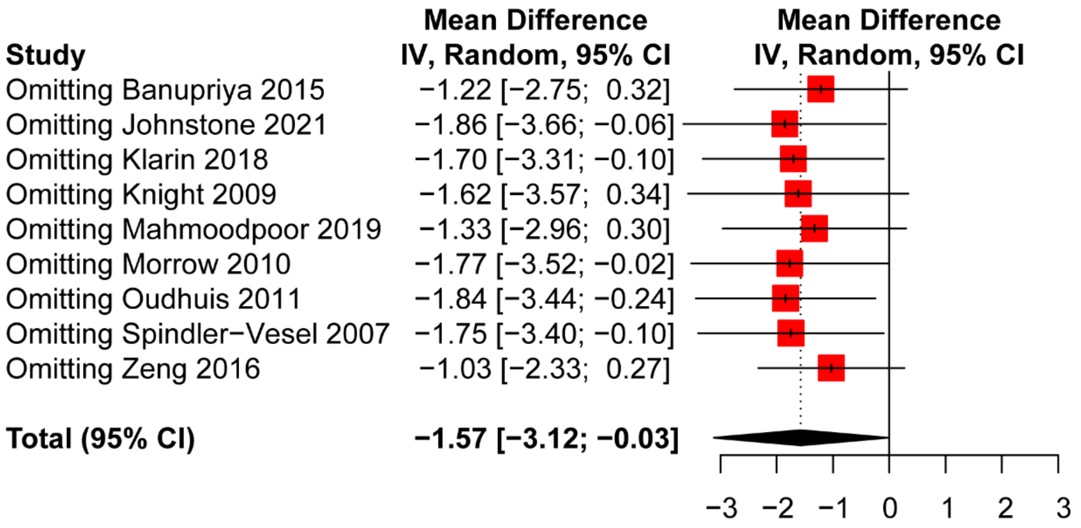

Figure 3: Sensitivity analysis for duration of mechanical ventilation

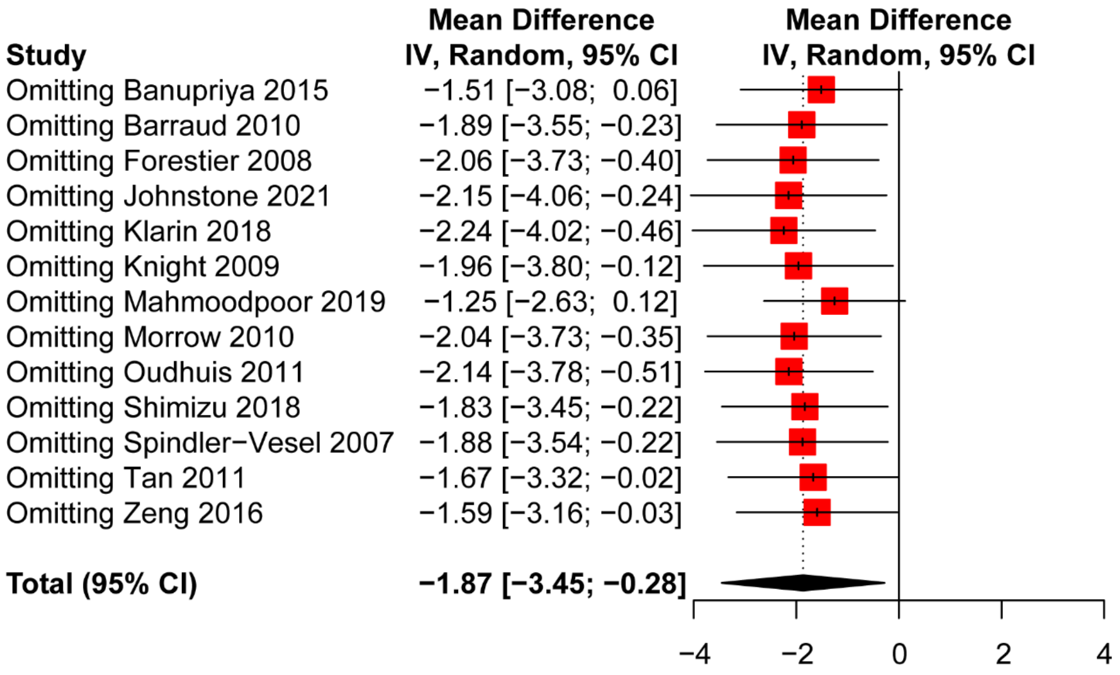

Figure 4: Sensitivity analysis for length of stay in intensive care unit

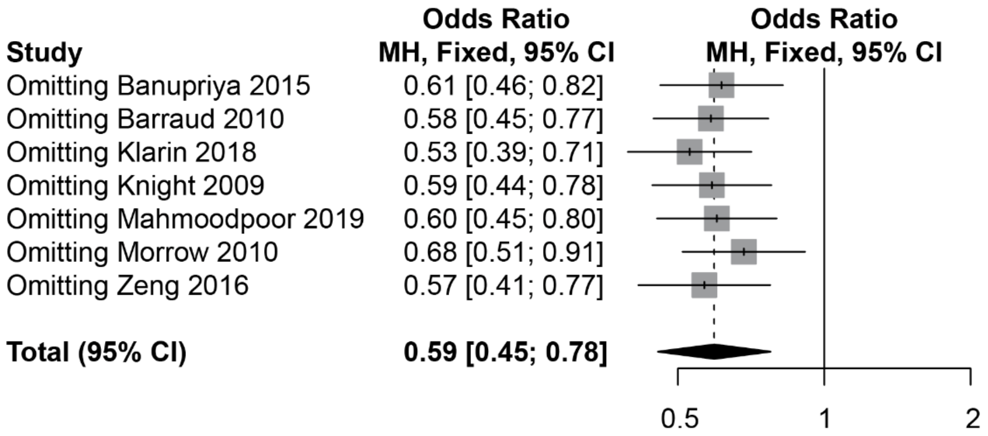

Figure 5: Sensitivity analysis for incidence of bacterial colonization

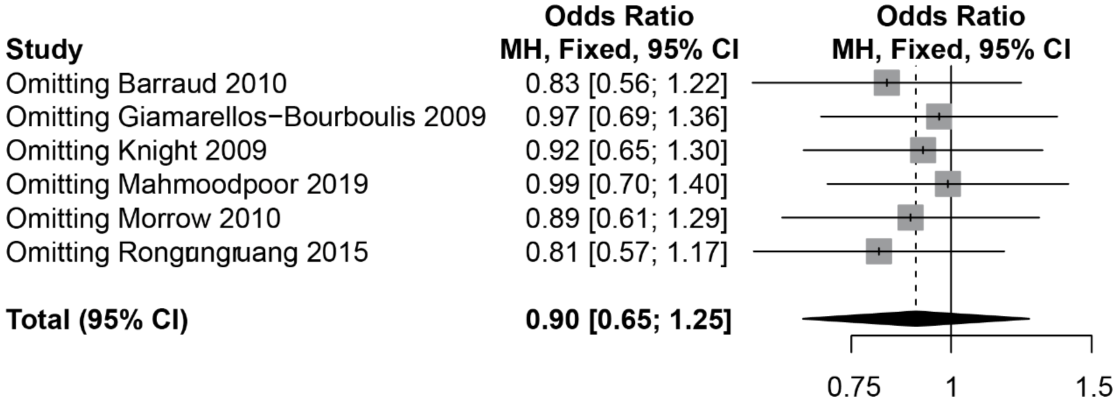

Figure 6: Sensitivity analysis for incidence of diarrhea
